# Physiology mechanisms of exercise for PTSD: a narrative review

**DOI:** 10.3389/fpsyg.2025.1483523

**Published:** 2025-01-27

**Authors:** Hongding Dong, Zhiyi Lin

**Affiliations:** ^1^Physical Education Institute of Jimei University, Xiamen, China; ^2^School of Physical Education and Sport Science, Fujian Normal University, Fuzhou, China

**Keywords:** post-traumatic stress disorder, Exercise intervention, non-pharmacological therapy, exercise and mental health, physiological mechanisms

## Abstract

In at-risk societies, the population of post-traumatic stress disorder (PTSD) incidence is gradually expanding from veterans to the general public. In the face of the high incidence of PTSD, exercise therapy, as an economical and maneuverable treatment, has not received the attention it deserves. In this paper, the literature on PTSD symptom improvement through comb-climbing exercise interventions found that performing long-term exercise can achieve significant improvement in PTSD symptoms by modulating the central nervous system, autonomic nervous system, and immune system at the physiological level. Aerobic exercise (running, walking) is beneficial to the central nervous system and immune system; anaerobic exercise positively affects the autonomic nervous system, including resistance or strength endurance training; yoga, which focuses on flexibility and balance training, has a positive effect on the immune system. Future research should explore the neutral and negative effects and mechanisms of exercise on PTSD interventions. Expand more empirical studies in special occupational populations. And implement longitudinal intervention studies with PTSD patients to gain an in-depth understanding of PTSD intervention effects.

## 1 Introduction

Post-traumatic stress disorder (PTSD) is a mental disorder triggered by traumatic events such as violence, natural disasters, or the death of a loved one. These events result in both psychological and physiological suffering (Maercker et al., 2022; Yehuda et al., 2015). PTSD often causes significant negative psychological consequences for individuals, their families, and society, including depression, substance abuse, and domestic violence (Emily, 2014). Studies reveal that approximately 70% of adult women in the United States have been exposed to a serious trauma (Resnick et al., 1993). The World Mental Health Surveys indicated that physical or sexual violence elevates the risk of developing PTSD (Liu et al., 2017).

In response to the high global prevalence of PTSD, therapeutic approaches across psychiatry, clinical medicine, and psychology have been explored. Biomedical methods, such as transcranial magnetic and electrical stimulation, have emerged as key treatments for PTSD (Ressler et al., 2022; Rosson et al., 2022; Van Rooij et al., 2021; Petrosino et al., 2021; Boggio et al., 2010). However, due to limited public awareness of mental health and societal stigma, patients often avoid discussing their mental health, which may worsen their conditions and discourage them from seeking treatment (Klik et al., 2019). Studies show that exercise therapy for PTSD avoids the hospital environment of traditional treatments. Its discreet and participatory nature reduces the stigma associated with PTSD, often linked to medical interventions and societal prejudices (Blum et al., 2021).

Therefore, there has been a progressive inclination toward exercise intervention therapy, arguably becoming one of the foremost modalities in evidence-based PTSD treatment (Caddic and Nsmith, 2018). An earlier review article delved into potential mechanisms through which aerobic exercise enhances the central nervous system in PTSD patients (Hegberg et al., 2019), yet there exists no comprehensive review endeavoring to synthesize the mechanisms by which exercise impacts each physiological system. Thus, this present review endeavors to explore the mechanisms underlying the amelioration of PTSD through exercise, encompassing not only the effects of specific exercise modalities on the central nervous system but also their impacts on the autonomic nervous system and the immune system.

## 2 Methods

### 2.1 Search strategy

Using Web of science, PubMed, and SPORTDiscus, we searched for studies published from 2000-01-02 to 2024-06-01. Keywords used included (Post-Traumatic Stress Disorder OR Post-Traumatic Neuroses) AND (exercise OR physical activity OR training OR fitness) AND (mechanism).

### 2.2 Inclusion and exclusion criteria

**Figure 1:**
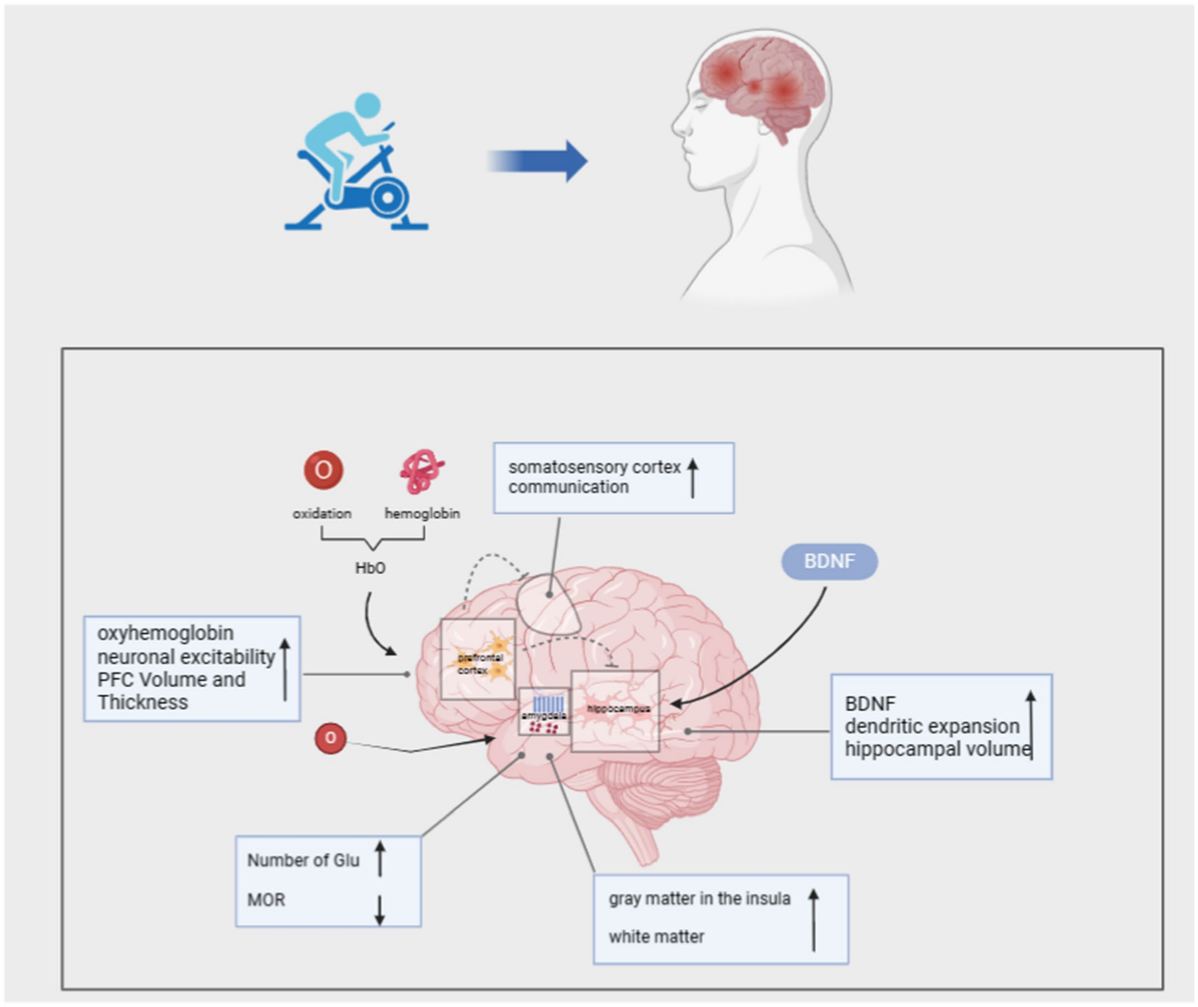
Mechanisms of CNS involvement in motor modulation of PTSD.

Only research and review literature published was included in this study. The topic of the included literature needed to be related to exercise and post-traumatic stress disorder or post-traumatic neuroses and physiologic mechanisms. Specifically, the review focuses on studies that: (1) address the exercise performance of individuals with PTSD, and (2) explicitly mention the physiological effects and mechanisms of exercise interventions for psychological disorder. In order to gain a more comprehensive understanding of the mechanisms of exercise interventions for PTSD, the subjects of exercise interventions were not limited. To further enhance the credibility and authority of the included literature, non-peer-reviewed articles, such as those from newspapers, news outlets, or conferences, were excluded. This decision ensures that the review incorporates only rigorously evaluated and methodologically robust studies. Non-peer-reviewed materials often lack academic rigor, detailed methodological descriptions, and transparency, reducing their reliability as references in academic reviews. For preventing subjectivity in literature screening, two researchers independently conducted the screening based on the criteria for inclusion and exclusion of literature (Figure 1).

**Figure 2:**
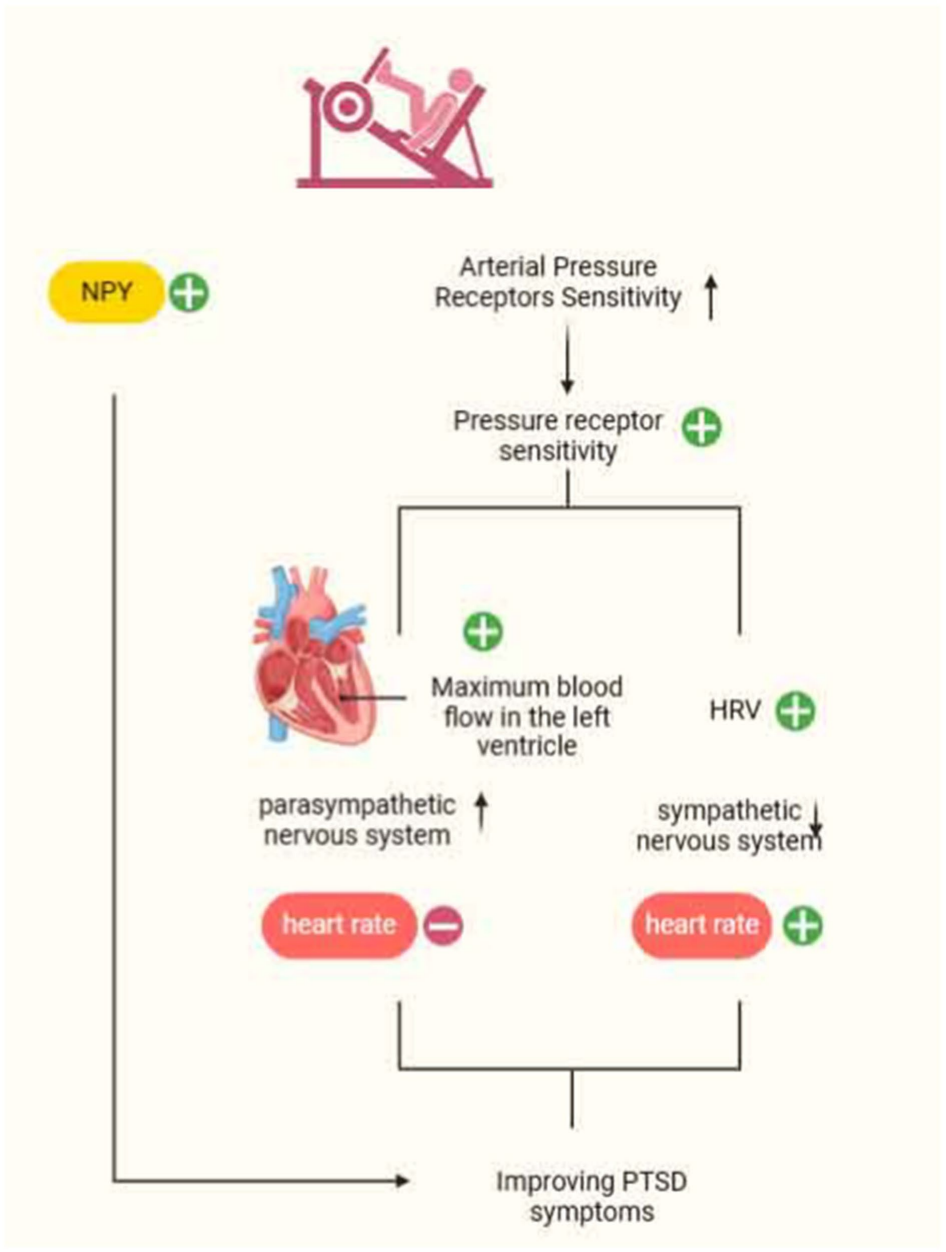
Mechanisms of autonomic nervous system involvement in motor modulation of PTSD.

After reviewing the articles, all authors reached a consensus to further screen the articles based on the following criteria: whether the studies were empirical, whether they employed exercise interventions, and whether they explained the physiological mechanisms underlying these interventions, among other considerations. The decision to include or exclude literature was made after agreement was reached. A total of 411 records were retrieved from the database. After removing duplicates, 221 records were reviewed for title, abstract, and article type to further exclude irrelevant literature. Most of the excluded literature was deemed irrelevant to the topic of this study. Relevant literature was manually identified by reviewing the references of selected papers. The full texts of the remaining literature were reviewed (Figure 2).

### 2.3 Quality assessment

**Figure 3:**
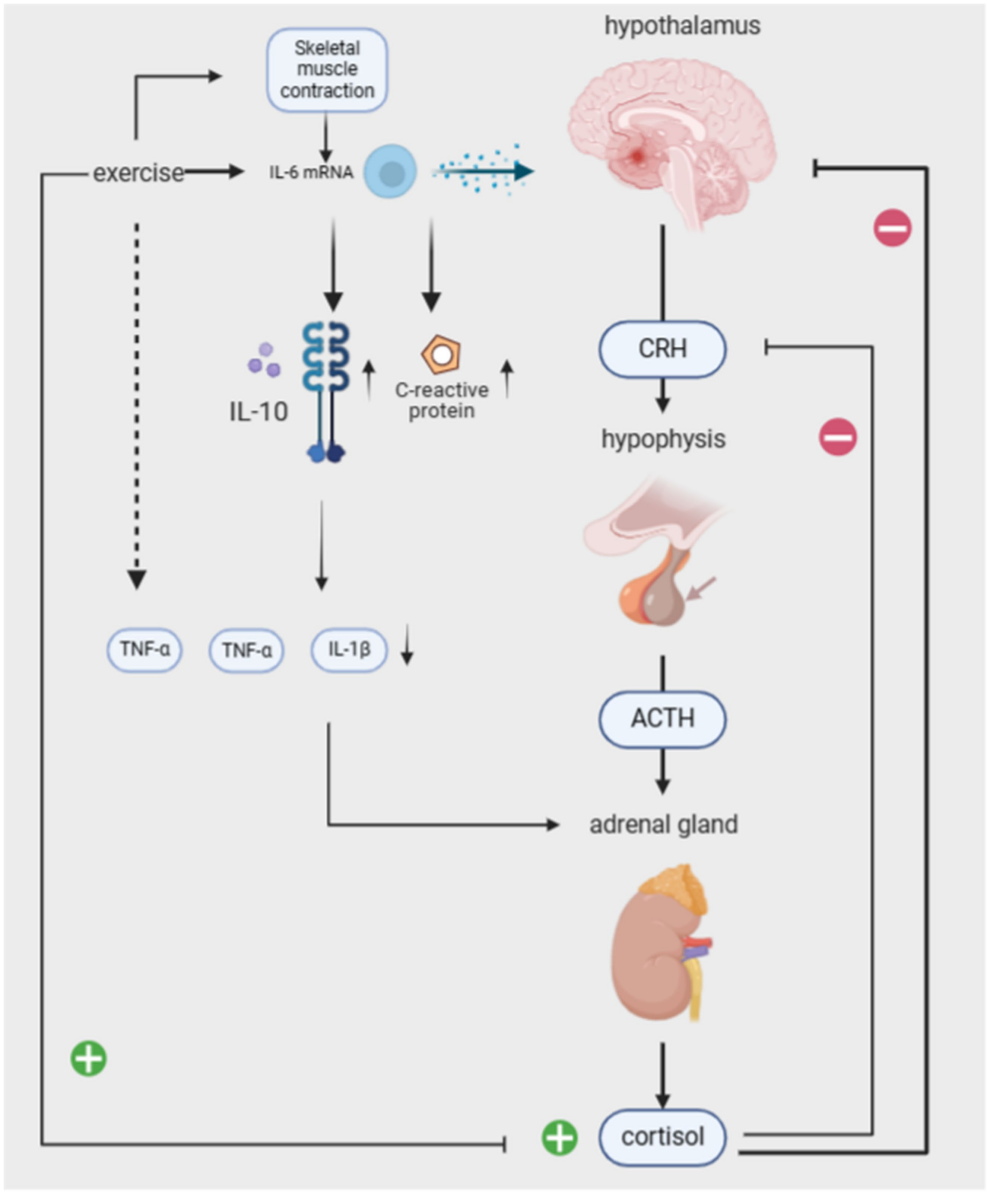
Mechanisms of immune system involvement in exercise modulation of PTSD.

A narrative review is a scholarly summary along with interpretation and critique. Narrative reviews contribute to prospective perspectives, it is concerned mainly with producing generalisable “facts” to aid prediction (Wiersinga et al., 2020). Wiersinga et al. (2020), for example, published a narrative review, cited in JAMA network, citation 3,460, explaining the pathophysiology of coronavirus disease and suggesting strategies to reduce transmission (Greenhalgh et al., 2018). Although narrative studies are subjective to judgment in screening the literature, it enables a more comprehensive review of research on exercise interventions for PTSD to inspire other researchers to further develop empirical research. To minimize the potential risk of selection bias, a methodological approach consistent with the PRISMA entries of this study was used, employing 15 entries from the 27-item checklist. Entries that were not relevant to the narrative review were deleted. Our adherence was limited by the nature of narrative reviews, which differ from the systematic and meta-analytical scope of PRISMA, particularly in areas of synthesis of results, statistical analysis, and bias assessment (Figure 3).

The average quality score of the articles included in this review was 8.03 (±1.29). Of these, 10.06% had lower scores, 33.3% were of medium quality, and 56.1% were of high quality. According to the scoring criteria, the mean score fell within the high-quality range. This indicates that the overall quality of these studies was high and satisfactory in terms of study design and data support.

### 2.4 Data extraction

The mechanisms relevant to this review were identified by reading the literature and discussing among the co-authors. After a comprehensive assessment of the included literature by the co-authors, there were 72 documents with data extracted on topics relevant to this review. The included literature spans from 1996 to 2023 and covers various types of exercise interventions, including yoga, running, walking, and strength training. The populations studied in these interventions include veterans, adult females, adult males, and older adults.

## 3 Physiologic mechanisms of exercise improvement in PTSD

### 3.1 Exercise modulates central nervous system interventions for PTSD

Experimental findings indicate that PTSD is linked with structural and functional alterations in brain regions such as the amygdala, hippocampus, cingulate cortex, hypothalamus, dorsolateral prefrontal cortex, and ventral prefrontal cortex (Scott et al., 2015). These brain regions perform vital functions crucial to daily life, including emotional processing, working memory, behavioral inhibition, and cognitive flexibility (Woon et al., 2017). Upon a diagnosis of PTSD, individuals often experience dysfunctional executive functions, manifested by cognitive decline, working memory loss, and other impairments. Recently, exercise interventions have demonstrated positive effects on the central nervous system, offering the academic community a fresh perspective on PTSD interventions.

#### 3.1.1 Prefrontal

Neural pathways in numerous cortical regions converge at the frontal lobes during the execution of high-level cognitive tasks involving executive functions. The Prefrontal Cortex (PFC) is crucial for integrating and regulating cognitive and emotional behaviors, serving as a key region facilitating adaptation to stressful stimuli (Cerqueira et al., 2008). Neuroimaging studies consistently demonstrate decreased activity in the medial prefrontal cortex (mPFC) among PTSD patients, impairing fear extinction compared to individuals without PTSD (Dahlgren et al., 2017). During working memory tasks, individuals exhibit increased activity in the left dorsolateral prefrontal cortex (DLPFC). Conversely, individuals with PTSD display significantly reduced activity in the DLPFC, leading to impaired memory for everyday speech (Clark et al., 2003).

Numerous studies have demonstrated that aerobic exercise enhances PFC activity. Loprinzi et al. conducted a study wherein a walking intervention was administered to patients with PTSD. They observed that moderate-intensity walking increased PFC activity, leading to elevated levels of oxygenated hemoglobin (HbO) in the left PLPFC post-exercise (Loprinzi et al., 2022). Oxygenated hemoglobin (HbO) delivers oxygen to neuronal tissues, promoting neuronal activity and enhancing communication between the PFC, somatosensory cortex (SMC), and hippocampus. This process effectively enhances the patient’s memory capacity and facilitates memory formation and retrieval. Research indicates that a 3-month muscle strength training and aerobic exercise intervention, particularly high-intensity aerobic exercise, can augment prefrontal lobe (PFC) volume and thickness in PTSD patients, resulting in cognitive and neurological improvement effects (Soshi et al., 2021). Furthermore, during exercise, muscle activation and the activation of muscle spindle waves trigger action potentials that propagate to the spine and brainstem, ultimately enhancing neuronal excitability in various memory-related brain structures (Gao et al., 2022).

#### 3.1.2 Hippocampus

The hippocampus is involved in cognitive and emotional regulation, and it is particularly vulnerable to stress in individuals with PTSD, leading to potential structural alterations. Research has shown that individuals with PTSD often exhibit a reduced hippocampal volume, which may contribute to the development of additional psychiatric comorbidities (Childress et al., 2013). Additionally, chronic stress diminishes the expression of brain-derived neurotrophic factor (BDNF) and impairs individual neuroplasticity, resulting in diminished spatial learning and memory, as well as compromised emotional regulation (Duman and Monteggia, 2006; Bonne et al., 2008). Within the hippocampus, neurogenesis encompasses processes such as cell proliferation, survival, migration, and neuronal differentiation, which contribute to neuroplasticity and enhance stress adaptation. Consequently, targeting neurogenesis is a common strategy for intervention in PTSD patients (Smith et al., 2010; Gujral et al., 2017). In this regard, experiments have shown that treadmill exercise is an important means of increasing the amount of tissue in the hippocampus, which restores neuroplasticity, inhibits cell death, and ultimately improves the symptoms of PTSD (Seo et al., 2019). Some researchers have also suggested that the increase in hippocampal volume promoted by aerobic exercise interventions is due to elevated levels of BDNF, which acts as a neurogenic mediator and contributes to dendritic expansion, which causes the hippocampus to increase in volume and thus promotes improvement in memory (Erickson et al., 2011). Specifically, exercise induces BDNF, stimulates neurogenesis, and increases neuroplasticity (Duman, 2005; Firth et al., 2018). By implementing a 30-day exercise intervention in mice, it was found that exercise mice had significantly higher levels of BDNF expression compared to control mice, which in turn resulted in enhanced spatial learning and memory. In particular, the exercise-induced increase in lactate levels is a key node in the induction of BDNF expression, which provides strong evidence for the “exercise-hippocampus-PTSD” mechanism of exercise therapy (El Hayek et al., 2019). Based on these basic experiments, further research in the academic community suggests that the degree of improvement in BDNF levels after aerobic exercise is mainly dependent on the intensity of exercise; moderate-intensity and low-intensity exercise are difficult to cause significant changes in BDNF, and moderate-intensity and high-intensity exercise can increase the level of BDNF and achieve the effect of improving cognitive function (Griffin et al., 2011; Chang et al., 2017). In conclusion exercise modulates the hippocampus and thus improves spatial memory in PTSD patients.

#### 3.1.3 Amygdala

The amygdala plays a crucial role in arousal, emotional stimulation, and the regulation of emotional and affective pain systems. Specifically, the basolateral amygdala (BLA) and the central nucleus (CEA) are critical for the acquisition of fear and extinction memories. Damage to the BLA disrupts fear memories, making individuals hypervigilant to threatening cues (Terburg et al., 2012; Giustino and Fmaren, 2015). Additionally, inhibitory neurons in the amygdala regulate fear output and are crucial for modulating mood and emotional distress (Kami et al., 2020). A study on autonomous running in model mice found a significant increase in glutamate (Glu) neurons in the BLA, which suppressed negative emotions such as pain, fear, and anxiety, while also stimulating pleasurable emotions (Sippel et al., 2018). However, as traumatic events recur, patients often over-suppress pain, resulting in a high pain threshold. This pattern of high-threshold responses to emotional stimuli manifests in PTSD patients as “emotional numbing,” characterized by a lack of love and happiness and an inability to respond to positive events (Liberzon et al., 2007). It has been found that pain and emotion inhibition is modulated by the amygdala and ventral striatum. When PTSD patients experience stress, the body releases endorphins to reduce pain, leading to an increase in *μ*-opioid receptor (MOR) levels and a lack of MOR downregulation (Korem et al., 2022). This results in heightened emotional inhibition and a slowed amygdala response, preventing timely and appropriate emotional responses to stress and hindering the implementation of emotional regulation strategies (Smith and Lyle, 2006).

Several studies have shown that peak oxygen uptake (VO2peak) after aerobic exercise correlates with a greater decrease in MOR binding in the ventral striatum, with higher exercise volumes demonstrating the ability of regular exercise to reduce the anti-injury effects of mu-opioid receptors in mice (Felmingham et al., 2014). Additionally, studies found that ventral striatal network reward processes were impaired in the amygdala of patients with PTSD and that aerobic exercise interventions effectively enhanced ventral striatal network reward function (Sacheli, 2019; The and Cortex, 2012). In summary, aerobic exercise can alleviate PTSD symptoms by down-regulating MOR and enhancing emotional and behavioral responses.

Conversely, the anterior insula (AI) plays a crucial role in processing emotions, including social emotions such as empathy and sympathy, and interpersonal functions like cooperation (Lamm and Singer, 2010). It is also involved in the assessment and expression of specific individual emotions, such as happiness, sadness, fear, and disgust (Schmitt et al., 2020). Evidence suggests that the functional connectivity between the amygdala and insula is altered in PTSD patients, manifesting as abnormal connectivity. High-intensity exercise has been shown to improve functional connectivity between the left amygdala and right anterior insula, thereby increasing positive emotions (Villemure et al., 2014). Villemure et al. (2014) demonstrated that cold pain tolerance in the left and right insula of individuals is positively correlated with gray matter (GM) volume. After a yoga intervention, participants exhibited increased insular cortex GM following long-term yoga practice, and white matter (WM) extending along the anterior and posterior aspects of the left insular GM also showed higher integrity, suggesting that enhanced intra-insular connectivity improves pain tolerance (Hill et al., 2018).

Additionally, exercise impacts the endogenous cannabinoid system (ECS), which is primarily expressed in the amygdala, in PTSD patients. The ECS includes components such as AEA, 2-AG, FAAH, and CB1r (Crombie et al., 2021). The ECS modulates cognitive and emotional responses, potentially playing a significant role in PTSD pathology. ECS deficiency may lead to increased stress susceptibility, contributing to trauma-related psychiatric disorders. Some experiments have indicated that moderate-intensity aerobic exercise can markedly increase circulating concentrations of AEA (N-arachidonylethanolamine), 2-AG (2-arachidonoylglycerol) and OEA (oleoylethanolamide) in PTSD patients, leading to the alleviation of fear and anxiety symptoms (Crombie et al., 2018).

Exercise presents a promising avenue for modulating the central nervous system in PTSD patients. By targeting regions such as the prefrontal cortex, hippocampus, and amygdala, exercise enhances neuroplasticity, regulates emotional processing, and improves cognitive function. These adaptations are critical in mitigating the core symptoms of PTSD, such as intrusive memories and emotional dysregulation. Notably, improvements in the central nervous system also exert downstream effects on the autonomic nervous system, creating a synergistic influence on overall stress regulation.

### 3.2 Exercise modulation of the autonomic nervous system intervention for PTSD

The autonomic nervous system (ANS) comprises sympathetic and parasympathetic nerves, with neuropeptide Y (NPY) serving as a sympathetic neurotransmitter primarily found in the periphery. NPY is involved in regulating stress responses in individuals. High activation of sympathetic nerves leads to the release of large amounts of NPY, resulting in increased blood pressure, inhibition of the vagus nerve, and the maintenance of energy balance. Studies have demonstrated decreased resting plasma NPY levels in veterans with PTSD, which diminishes their anxiolytic effects (Rämson et al., 2011; Scioli et al., 2020). To harness the function of NPY and alleviate PTSD symptoms, Rämson investigated the use of exercise training to enhance NPY function. Maximum-load exercise was identified as a potential mediator of PTSD symptom improvement in a study involving 90 veterans who underwent 3 months of progressive aerobic exercise. In addition, resistance training also has an effect on NPY release, enhancing NPY synthesis under stress conditions and increasing anxiolysis levels to ameliorate PTSD symptoms (Levine et al., 2014). Paradoxically, chronic stress-induced over-activation of the negative feedback regulation of NPY and the “exercise-NPY” production mechanism can lead to excessive NPY release into adipose tissue, resulting in obesity and metabolic syndrome (Levine et al., 2014). Thus, the challenge lies in enhancing anxiolysis levels while mitigating related comorbidities, a topic that warrants further investigation in subsequent studies.

Evidence suggests that resting parasympathetic nervous system (PNS) activity decreases and sympathetic nervous system (SNS) responses increase in PTSD patients under mental stress. Overactivation of the SNS during an individual’s encounter with psychological stress leads to abnormal bodily reactions, potentially resulting in diseases such as chronic heart failure, obesity, and hypertension. One potential mechanism influencing this process is a decrease in arterial baroreflex sensitivity (BRS) (Park et al., 2017). Arterial pressure receptors buffer arterial blood pressure fluctuations caused by physiological and psychological stress, playing a crucial role in immediate blood pressure control. Additionally, arterial pressure receptors can inhibit SNS activity, helping maintain blood pressure close to a set value. Studies have found that years of strength endurance training can markedly reduce heart rate, increase arterial pressure receptor sensitivity, and consequently assist individuals in regulating SNS abnormalities. Specifically, changes in parameters such as the Heather index, index of contractility (IC), acceleration index (ACI), and time to achieve maximal contractile strength of the left ventricle provide robust evidence of the increase in parasympathetic stimulation induced by exercise. Furthermore, spectral analysis showed that strength-endurance exercise led to a higher frequency of the heart rate variability (HRV) spectrum and a reduction in sympathetic nerve activity (Kowalik et al., 2019). Thus, arterial pressure receptor sensitivity is likewise one of the important targets for reducing cardiovascular complications in PTSD patients. In summary, exercise has the potential to regulate the autonomic nervous system, thereby alleviating PTSD symptoms. This regulatory effect also lays the foundation for understanding its broader influence on physiological systems, including the immune system (Kenney and Ganta, 2014).

### 3.3 Exercise modulates the immune system to intervene in PTSD

Previous studies have demonstrated various interactions between the autonomic nervous system and the immune system, which underpin chronic inflammatory diseases, such as organ inflammation, pain, cardiovascular involvement, and fatigue (Bellocchi et al., 2022). PTSD is suggested to dysregulate the biological pathways of the autonomic nervous system (ANS) and the HPA axis, resulting in a pro-inflammatory state that triggers chronic low-grade inflammation (Neigh and Nali, 2016; Speer et al., 2018). During traumatic events in PTSD patients, cortisol levels decrease while immune system inflammation increases, resulting in a 2- to 3-fold rise in plasma concentrations of pro-inflammatory cytokines IL-1, IL-6, and TNF-*α*. This continuous elevation of inflammatory factors in the body, coupled with the failure of cortisol’s immune-suppressive and anti-inflammatory effects, contributes to chronic low-grade inflammation symptoms (Quinones et al., 2020; Pivac et al., 2023). Additionally, inflammation may predispose individuals to PTSD or even serve as the biological basis for triggering PTSD (Smid et al., 2015). Generally, both pro-inflammatory and anti-inflammatory cytokines are produced in response to *in vitro* stimuli, and their balance in the body determines the development of a pro- or anti-inflammatory environment. Therefore, both pro-inflammatory and anti-inflammatory cytokines may contribute to the development of PTSD symptoms. Pro-inflammatory factors directly adversely affect memory function and neuroplasticity. Severe psychological trauma stimulates several components of the immune system, with IL-1 playing a significant role, resulting in increased expression of pro-inflammatory cytokines in various brain regions such as the hippocampus, hypothalamus, and brainstem. Additionally, peripheral immune cells produce pro-inflammatory cytokines that affect various brain regions through humoral and neural pathways (Smid et al., 2015). Anti-inflammatory cytokines produced by exercise serve as a buffer against an overly pro-inflammatory environment. IL-6 mRNA is elevated during exercise through skeletal muscle contraction, which stimulates the release of the anti-inflammatory cytokines IL-10 and IL-1 receptor antagonist (IL-1ra), IL-10 does minimize inflammation-induced tissue damage, and IL-1ra inhibits the pro-inflammatory effects of TNF-a and IL-1β effects (Gopal et al., 2011; Fischer, 2006). This also stimulates the release of cortisol from the adrenal glands (which has an anti-inflammatory effect). IL-6 elevation due to exercise is evident in promoting an anti-inflammatory environment in the body by stimulating various cytokines. Trauma-induced reductions in the anti-inflammatory cytokine IFN-*γ* may also compromise cellular immunity. One study investigated the immune system of two groups of subjects after exposure to exam stress. It found that as stress increased, the serum IFN-*γ* level significantly decreased in the control group, while the difference in serum IFN-γ level between the yoga group and the pre-stress level was not statistically significant. It can be seen that yoga has a certain buffering effect on cellular immune damage.

PTSD is associated with cardiovascular disease (CVD) through complex mechanisms, with inflammation playing a key role in the pathogenesis of CVD. Inflammation is the immune system’s response to injury, aimed at removing pathogens from the infected area and promoting healing (Starkie et al., 2003). C-reactive protein (CRP) is an acute-phase protein and a biomarker of inflammation, regulated by IL-6 and other pro-inflammatory cytokines (Gleeson et al., 2011). Some studies have shown that the intrusive and avoidant symptoms of PTSD are significantly associated with CRP, indicating that PTSD may lead to immune system dysregulation (elevated CRP levels). Prolonged dysregulation can result in health risks such as obesity, cardiovascular disease, and other health problems (Alfaddagh et al., 2020).

A study of older adults compared the effects of aerobic exercise versus flexibility exercise on inflammation and mental illness. The intervention consisted of 45 min of aerobic exercise, three times a week for 10 months. Both types of exercise were found to improve depression and increase optimism. Aerobic exercise had the greatest effect on inflammation, with a significant reduction in CRP at the end of the intervention (Canetti et al., 2014). Additionally, lowering the respiratory rate can achieve a reduction in CRP and pro-inflammatory cytokines, thereby alleviating symptoms of hyperarousal (Kohut et al., 2006).

The central nervous system (CNS) and the sympathetic nervous system (SNS) interactively maintain the body’s adaptive physiological functions. The hypothalamic–pituitary–adrenal axis (HPA), the body’s major stress system, interacts with the SNS. The hypothalamus secretes corticotropin-releasing hormone (CRH), stimulating the pituitary gland to release adrenocorticotropic hormone (ACTH). ACTH stimulates the adrenal glands to release cortisol, which in turn acts on the hypothalamus and pituitary gland, providing negative feedback to regulate the system. According to the cross-stressor adaptation hypothesis, exercise training enhances the physiological stress response system’s adaptation, promoting cross-stressor tolerance. It even reduces sensitivity to non-exercise stressors (Kelly et al., 2018).

Studies have reported differences between the HPA axis in PTSD patients and the non-PTSD population, suggesting that risk factors for HPA axis dysregulation mainly include CRH overactivity and low cortisol levels (Sothmann and Facsmbuckworth, 1996). Excessive activation of CRH, a regulator of the HPA axis, amplifies the individual’s fear response. Li et al. found that long-term regular running exercise attenuates the response to exogenous CRH and raises basal cortisol levels, improving the functional state of the HPA axis. Related studies have also shown that 8 weeks of stretching and balancing exercises, combined with breathing and positive thinking training, can significantly increase basal serum cortisol concentrations, normalize cortisol levels, and improve PTSD symptoms (Dunlop and Wwong, 2019; Kim et al., 2013). Balasubramaniam et al. found that yoga interventions can modulate the HPA axis and increase BDNF, thereby reducing inflammation and lowering stress and anxiety (Balasubramaniam et al., 2012).

## 4 Conclusion

Although a large number of studies have been conducted to illustrate the physiologic mechanisms of PTSD symptoms, few studies have explored the complex relationship between exercise and PTSD symptoms. Exercise is now recognized as an effective intervention for improving PTSD. This review provides an overview of the physiological mechanisms by which exercise alleviates PTSD symptoms. Key mechanisms include promoting prefrontal oxygenation, enhancing neurogenesis and cell proliferation in the hippocampus, and modulating the activation of the amygdala and the stress-related HPA axis. Additionally, exercise contributes to balancing the immune response by enhancing anti-inflammatory processes while regulating pro-inflammatory cytokines.

Furthermore, exercise interventions help normalize autonomic nervous system function, which is crucial for reducing hyperarousal and improving cardiovascular health in PTSD patients. These multifaceted benefits underscore the importance of incorporating exercise into therapeutic strategies for PTSD.

In summary, this narrative review explores a topic of high relevance to public health. The current scientific evidence strongly supports the conclusion that therapeutic interventions for PTSD should focus not only on alleviating psychiatric symptoms but also on addressing related issues such as sleep disorders and chronic inflammation. Future research should aim to understand the mechanisms by which exercise can be combined with other interventions.

Several limitations of the studies included in this review should be considered. There are sex differences in physiological structure, neurotransmitters, and sex hormones in humans. For instance, sex differences in brain structure affect memory and emotional regulation (Compère et al., 2016). Differences in neurotransmitters also lead to variations in behavioral motivation, impulse control, and problem-solving strategies. Therefore, the mechanisms and effects of exercise interventions for PTSD patients may differ by gender, but current research has not explored these differences. Furthermore, personalized exercise intervention programs for PTSD patients based on gender are rarely seen. Age differences in PTSD patients should also be considered. Additionally, the mechanisms of exercise interventions for PTSD require further analysis from a micro perspective. The effects of exercise interventions on various neurotransmitter and hormonal systems in PTSD patients remain to be further explored, such as glutamatergic and GABAergic systems. Although it has been demonstrated that exercise interventions can influence the central nervous system regions highly associated with PTSD (e.g., prefrontal cortex, hippocampus, and amygdala), these findings are primarily based on animal studies or preliminary human research. They have not yet been effectively translated into clinical applications. Additionally, whether exercise interventions induce interactions between these systems requires further investigation. In addition, studies generally use small sample sizes for exercise interventions, limiting the external validity of the results.

Most empirical studies have demonstrated that aerobic exercises, such as running and walking, positively impact the central nervous and immune systems of individuals with mental illnesses. Anaerobic exercises primarily benefit the autonomic nervous system, with interventions typically involving resistance or strength endurance training. Yoga, a form of flexibility and balance training, has also been shown to enhance the immune system of PTSD patients, thereby alleviating PTSD symptoms. However, the physiological mechanisms underlying other types of exercise and combinational exercise, remain underexplored. Consequently, future research should prioritize high-quality randomized controlled trials to further investigate these mechanisms. Also, in order to gain insight into the benefits of exercise interventions for mental disorders. More longitudinal studies are needed to monitor the effects of exercise interventions for PTSD in real time. To understand the impact of different lengths of exercise programs on the effectiveness of improving PTSD symptoms. Additionally, incorporating innovative research methods, such as ecological momentary assessment (EMA), could enable individuals to implement interventions directly in their daily lives.

To further guide future researchers and practitioners in implementing exercise interventions in clinical and community settings, we will briefly introduce several beneficial exercise intervention programs for PTSD. It is worth noting, however, that this article does not focus on a specific exercise intervention program. Therefore, this section provides a concise overview of the current mainstream exercise intervention programs. The programs are provided for reference. Previous research has identified several types of exercise interventions for PTSD, including greenfield exercise, aerobic exercise, mixed exercise, and combined interventions. For instance, rock climbing (a type of green exercise) and aerobic exercise have been shown to reduce anxiety symptoms and improve depression and worry in PTSD patients (Wheeler et al., 2020; Crombie et al., 2021). Fishing has also been shown to significantly improve PTSD. A fly-fishing intervention program for veterans with PTSD resulted in significant improvements in attention and positive emotional states, with sustained reductions in depression, anxiety, and physical stress symptoms (Vella et al., 2013). The combined intervention of meditation and aerobic exercise also has benefits in addressing trauma-related memory and cognition by modulating both branches of the autonomic nervous system-the sympathetic and parasympathetic systems (Shors et al., 2018). Regarding exercise intensity and duration, long-term interventions (lasting more than 5 weeks) are recommended for PTSD patients, with a session duration of 30 to 60 min. Such interventions can increase the concentration of BDNF in the patient’s serum. Implementing moderate to high-intensity exercise interventions for PTSD patients can increase circulating levels of the endocannabinoid system (AEA and 2-AG). This can reduce subjective anxiety and stress while enhancing the individual’s tolerance to stressors (Griffin et al., 2011). These studies have provided valuable insights for the development of future exercise intervention programs.

Despite the convenience advantages of exercise over treatments like medication and exposure therapy, implementing an intervention program also requires relevant personnel to provide comprehensive logistical support to patients. For instance, the government should consider expanding natural environments to provide spaces where patients can engage in green sports. In the case of combined exercise interventions, physical trainers and physicians should collaborate to develop individualized intervention plans for patients. One example is the combination of aerobic exercise and medication. Researchers could develop software for exercise interventions, enabling patients to access treatment programs tailored to various PTSD symptoms by inputting their symptoms into the software.

Currently, there has been no systematic empirical research on whether exercise exacerbates PTSD or whether it produces an inability to alleviate PTSD symptoms. Hegberg mentions in his study that no negative effects of exercise on PTSD have been found (Hegberg et al., 2019). But this point of view is not entirely accurate. In another study, interviews with clinical workers found that asking patients with PTSD to exercise outdoors means that it requires them to leave the indoor environment where they feel safe, however this outdoorness may cause anxiety and exacerbate PTSD (Zhu et al., 2024). In addition to this, it has also been suggested that the physical sensations and hyperarousal symptoms (e.g., increased respiration, heart rate, sweating) of strenuous exercise may lead people with hyperarousal symptoms to avoid strenuous exercise (Whitworth et al., 2017). Thus, exercise as an intervention for PTSD should be approached dialectically. We recommend that patients closely monitor their physical and psychological status both before and after engaging in exercise interventions. This can include tracking changes in mood, energy levels, and any physical discomfort or improvement. If symptoms worsen or unexpected side effects occur following an exercise intervention, it is crucial for patients to seek timely assistance from a mental health professional. Early intervention can prevent further exacerbation of symptoms and ensure that the exercise program is adjusted to better suit the patient’s needs. Additionally, collaboration with healthcare providers can help tailor the intervention to align with the patient’s individual conditions and recovery goals.

Special populations, such as individuals engaged in professional occupations, have not yet received sufficient attention. Some studies have suggested that athletes, one of professional occupations, may exhibit higher levels of PTSD compared to the general population (Aron et al., 2019). The exercise intervention approach targeted at athletes who have PTSD, will significantly differ from that applied to military personnel or the general population. Currently, most interventions are performed in experiments, which excludes the possibility of some sports injuries occurring. When patients return to exercise in natural contexts, they are bound to experience uncertainties in the exercise environment, such as injuries and competition failures, and develop PTSD. therefore, further exploration of the mechanisms of exercise interventions for PTSD in life situations is necessary. In Toyoda et al.’s study, it was mentioned that some people with long-term physical activity may still suffer from PTSD (Toyoda et al., 2023). Therefore, the academic community should focus on the specificity of exercise interventions for PTSD in athletes, particularly regarding how to reduce PTSD when high-intensity exercise is performed daily (Yang et al., 2022). Currently, strategies for intervening with athletes include exposure therapy, expressive writing, and self-desensitization. Future intervention programs could integrate these therapeutic approaches with physical activities such as yoga, meditation, or martial arts.

As this study aimed to summarize the physiological mechanisms of exercise interventions for PTSD, the specific micro-mechanisms within the three physiological systems (central nervous system, autonomic nervous system, and immune system) were not examined in detail due to the scope of the study. Future research could investigate the micro-level effects of exercise interventions for PTSD, such as variations in intervention outcomes based on gender and age. The populations involved in this study include veterans, adult males, adult females and so on. For this population, PTSD symptoms mostly arise from facing casualties, killings, and miscarriages. These findings may not be representative of exercise interventions for all patients with PTSD in the general population. Variations in the etiology and presentation of PTSD across different populations may limit the generalizability of the results. For instance, adults with a history of specific trauma may respond differently to exercise interventions. And there may be a lack of explanatory power in this review when it comes to PTSD problems faced by athletes and drone bombing commanders. Therefore, more empirical research is needed in the future to explore the specificity of intervention mechanisms for different groups.
